# Insulin-like Growth Factor 1 (IGF-1) in Hair Regeneration: Mechanistic Pathways and Therapeutic Potential

**DOI:** 10.3390/cimb47090773

**Published:** 2025-09-18

**Authors:** Wang-Ju Hsieh, Wei-Yin Qiu, Ivona Percec, Tsong-Min Chang

**Affiliations:** 1 Schweitzer Biotech Company, Taipei City 114066, Taiwan; 2 Department of Clinical Application, Center for iPS Cell Research and Application (CiRA), Kyoto University, Kyoto 606-8507, Japan; 3 Division of Plastic Surgery, Department of Surgery, University of Pennsylvania, Philadelphia, PA 19010, USA; 4 Department of Applied Cosmetology, Hungkuang University, Taichung City 433304, Taiwan

**Keywords:** hair regeneration, IGF-1, growth factor, hair growth, hair follicle, alopecia, hair loss, exosome-based drug delivery, molecular pathways

## Abstract

IGF-1 (insulin-like growth factor 1) is a growth factor primarily secreted by dermal papilla cells on hair-bearing skin that stimulates hair follicle proliferation and vascularization, and promotes the transition to the anagen growth phase of the hair follicle by activating key pathways such as PI3K/Akt and MAPK/ERK. IGF-1 also inhibits apoptosis, prolongs the follicular growth phase, and boosts VEGF expression, which supports microcirculation and nutrient delivery to hair follicles. The combined effects of IGF-1 and other growth factors, including VEGF, KGF (FGF-7), and PDGF, further amplify its effects on follicular cell proliferation and tissue repair. IGF-1’s ability to regulate the hair growth cycle and its interactions with other signaling pathways make it a compelling therapeutic target for hair loss disorders. Both preclinical models and clinical evidence highlight IGF-1 as a promising therapeutic option for conditions like androgenetic alopecia (AGA), where IGF-1 levels are typically diminished. While topical IGF-1 treatments have shown efficacy and safety with minimal systemic absorption, additional research is needed to improve delivery methods, such as liposomal gels and exosome-based carriers, and to evaluate long-term effects.

## 1. Introduction

Hair is deeply tied to self-image and mental health, with hair loss (e.g., androgenetic alopecia) often linked to low self-esteem, social anxiety, and depression [1]. Moreover, hair loss can be associated with underlying health issues such as thyroid disease, diabetes, or lupus and hormone disruptions [1]. Environmental damage, especially UV exposure, also contributes to hair degradation by breaking down keratin [2], making UV protection a vital component of hair care.

This review highlights the key role of IGF-1 in hair regeneration. Secreted mainly by dermal papilla cells, IGF-1 promotes hair follicle cell proliferation, vascularization, and transition into the anagen (growth) phase through PI3K/Akt and MAPK/ERK signaling pathways. It also prevents follicular cell apoptosis and boosts VEGF expression, enhancing blood flow and nutrient delivery to follicles. Its ability to extend the anagen phase, stimulate follicle growth, and inhibit cell death has made IGF-1 a promising therapeutic candidate for addressing hair loss and promoting healthy hair growth [3]. Beyond its role in hair growth, IGF-1 plays widespread physiological roles [4]: supporting muscle and bone growth, improving heart function [5], enhancing insulin sensitivity, and aiding in metabolic regulation.

## 2. Role of IGF-1 in Hair Follicle Growth

The healthy structure of the hair follicle is a key element of hair growth. Thus, understanding the microanatomy and regulation of the hair follicle is essential for clinicians managing patients with hair disorders. Each hair follicle consists of the following functionally distinct regions: [6].

Dermal Papilla (DP): Located at the base of the hair follicle, the DP regulates the hair growth cycle by secreting various paracrine growth factors, such as VEGF (vascular endothelial growth factor), which promotes angiogenesis and nutrient supply [7].

Outer Root Sheath (ORS): Surrounding the hair shaft, the ORS provides protection and support, as well as multipotent stem cells involved in hair structure regeneration and repair.

Inner Root Sheath (IRS): Located inside the ORS, the IRS forms a sheath around the emerging hair shaft, directing its proper alignment and growth.

Sebaceous Gland: Connected to the hair follicle, it secretes sebum to lubricate the skin and hair, maintaining their health.

Matrix Area: Located above the DP, this area consists of rapidly proliferating cells (keratinocytes) that differentiate and produce the hair shaft. Melanocytes within the matrix contribute to hair color by producing melanin [8,9].

The above structures work together to maintain the hair follicle cycle, which comprises three main phases:

1. Anagen (growing) phase: Lasting 2–8 years on the scalp or 2 to 3 months on the eyebrows, anagen is characterized by active matrix–cell proliferation and melanogenesis while the hair bulb is tightly connected to the skin tissue [8,9]. Hair shaft elongation occurs during this stage, influenced by DP signals that regulate matrix–cell numbers [9]. Hair follicles in different body parts produce hairs of varying lengths, which is proportional to the duration of the anagen phase [9].
2. Catagen (transition) phase: A brief apoptotic process where keratinocytes and melanocytes undergo programmed cell death (apoptosis). At the end of this phase, the DP retracts toward the bulge, a critical interaction point for initiating the next cycle [8,9]. Failure of the dermal papilla to reach the bulge during this phase may halt the hair cycle, causing hair shedding, which has been observed in both humans and mice [9].

Telogen (resting) phase: Telogen phase allows communication between bulge stem cells and the repositioned DP to prime follicular re-entry into anagen phase by activation of a critical concentration of stem cells [8]. IGF-1 is a key regulator of the hair growth cycle by promoting the proliferation and differentiation of hair matrix–cells and keratinocytes [3]. IGF-1 is synthesized by DP cells and exerts its proliferative and anti-apoptotic effects on matrix keratinocytes and DP cells by binding to IGF-1R receptor, primarily through the PI3K/Akt and MAPK/ERK pathways [3]. Functionally, IGF-1 prolongs the anagen phase and delays catagen phase onset in human hair follicle organ cultures [10,11]. It also promotes the VEGF expression, enhancing angiogenesis around the hair follicle and metabolic support for actively growing follicles [12]. Importantly, IGF-1’s clinical relevance is illustrated in androgenetic alopecia (AGA), where decreased IGF-1 expression by DP cells in balding scalp regions correlates with follicular degeneration and anagen shortening [13]. Therapeutic strategies targeting IGF-1 pathways may thus hold promise in arresting or reversing follicular regression in AGA and other hair loss conditions.

## 3. IGF-1 Structure and Signaling in Hair Follicle Regulation

### Structure Overview of IGF-1

**Figure 1:**
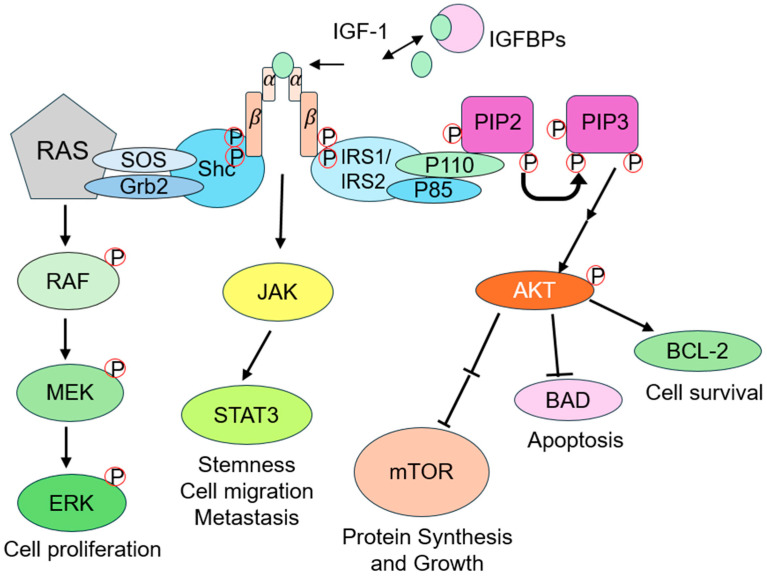
Diagram of the IGF-1 signaling pathway. IGF-1, regulated by IGF-binding proteins (IGFBPs), binds to the IGF-1 receptor (IGF-1R), a tetrameric receptor with two α and two β subunits. Ligand binding triggers receptor autophosphorylation (denoted by ‘P’) and recruits adapter proteins, including insulin receptor substrates 1/2 (IRS1/IRS2) and Shc. This initiates three key downstream pathways: (1) the PI3K-AKT pathway, where the p85 regulatory and p110 catalytic subunits convert phosphatidylinositol 4,5-bisphosphate (PIP2) to phosphatidylinositol 3,4,5-trisphosphate (PIP3), activating AKT to promote cell survival via BCL-2, inhibit apoptosis through BAD, and drive protein synthesis and growth via mTOR; (2) the MAPK/ERK pathway, mediated by SOS and Grb2, which activates RAS, followed by sequential phosphorylation of RAF, MEK, and ERK to stimulate cell proliferation; and (3) the JAK-STAT3 pathway, which enhances stemness, cell migration, and metastasis. Arrows indicate signaling flow, with arrow heads indicating stimulation and flat heads indicating inhibition. Circled P symbols highlighting critical phosphorylation events.

IGF-1 is a 70-amino acid-long polypeptide hormone stabilized by three disulfide bonds with a structure similar to insulin [14]. Its primary biological effects are mediated by binding to IGF-1R, a member of the tyrosine kinase receptor family composed of two α-subunits and two β-subunits [15]. Upon IGF-1 binding, IGF-1R undergoes autophosphorylation, activating downstream signaling pathways such as PI3K/AKT and MAPK that promote cell proliferation and differentiation while inhibiting apoptosis (Figure 1) [16]. The bioavailability and distribution of IGF-1 are tightly regulated by IGF-binding proteins (IGFBPs), which form high-affinity complexes with IGF-1. IGFBPs can inhibit IGF-1 activity by masking its receptor-binding domain or, conversely, potentiate its effects by facilitating local receptor engagement [17].

## 4. Key IGF-1 Signaling Pathways in Hair Follicle Regulation

### 4.1. PI3K/AKT Signaling Pathway

IGF-1R autophosphorylation following IGF-1 binding, PI3K is recruited and converts phosphatidylinositol 4,5-bisphosphate (PIP_2_) to phosphatidylinositol (3,4,5)-trisphosphate (PIP_3_), which in turn recruits and activates protein kinase B (AKT). AKT exerts proliferative effects by phosphorylating and inhibiting pro-apoptotic proteins such as Bad and Caspase 9 [18]. It also activates mTOR (mammalian target of rapamycin), which promotes protein synthesis, nutrient availability, and cell growth [19]. Additionally, AKT plays a key role in glucose and lipid metabolism, enhancing glucose uptake and stimulating glycogen synthesis, which may support the high metabolic demands of hair follicle cell proliferation [20].

### 4.2. MAPK/ERK Signaling Pathway

IGF-1R activation also triggers the RAS-RAF-MEK-ERK cascade via the adaptor protein complex Grb2/SOS. Once phosphorylated, ERK translocates into the nucleus to activate transcription factors, promoting cell proliferation and differentiation [21]. In the context of hair follicles, this pathway facilitates matrix–cell proliferation and follicular morphogenesis.

### 4.3. Other Signaling Pathways

IGF-1R can also activate the JAK/STAT pathway, particularly STAT3, which has been associated with tumorigenesis under certain conditions [21].

## 5. IGF-1 and Hair Follicle Function

In hair follicles, IGF-1 is mainly synthesized by dermal papilla cells, hair follicle matrix–cells, and dermal fibroblasts [22]. Its receptor IGF-1R is primarily located in the dermal papilla and matrix–cells of the hair follicle [10]. The proliferative effect of IGF-1 on hair follicle cells can vary by cell type. In dermal sheath cells, IGF-1 mainly acts through the PI3K/Akt pathway, which inhibits apoptosis and promotes cell survival and proliferation [23]. In contrast, in dermal papilla cells, IGF-1 preferentially signals through the MAPK pathway via the Shc-Grb2-SOS adaptor complex, leading to the RAS-RAF-MEK-ERK cascade activation and enhancing cell proliferation and differentiation [24]. These signaling effects contribute to the proliferation of epithelial matrix–cells, hair follicle cell differentiation, and follicular morphogenesis [10,25]. As a survival factor, IGF-1 prolongs the anagen phase by inhibiting apoptosis to support sustained hair shaft growth [26]. The energy demands of these processes are supported by IGF-1 pathway interaction with metabolic regulators such as mTOR and AMP kinase (AMPK), integrating energy availability with hair growth [26].

## 6. Regulation of IGF-1

IGF-1 activity levels of hair follicles are modulated by IGFBPs, in particular IGFBP-3 synthesized by dermal papilla cells [10,27]. By binding to IGF-1, IGFBPs may either inhibit IGF-1 by sequestering free IGF-1 or enhance its engagement with IGF-1R by bringing it closer to IGF-1R. Exogenous IGF-1 administration has been shown to upregulate IGFBP-3, altering local IGF-1/IGF-1R dynamics [27]. Such feedback mechanisms suggest a tightly regulated paracrine environment governing hair follicle response to IGF-1. Understanding the nuances of IGF-1 pathway activation in distinct follicular compartments may guide future interventions aimed at modulating hair growth.

## 7. IGF-1 Signal Transduction Mechanisms in Hair Follicles

**Figure 2:**
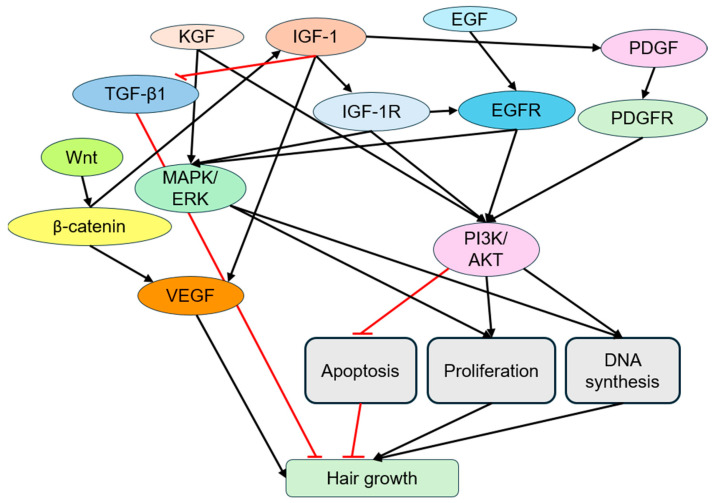
Diagram depicting the interplay of IGF-1 and other growth factors in hair growth regulation. IGF-1 binds to its receptor (IGF-1R), integrating with signaling from epidermal growth factor (EGF) via EGF receptor (EGFR), PDGF via PDGF receptor (PDGFR), keratinocyte growth factor (KGF), transforming growth factor-β1 (TGF-β1), Wnt pathway (via β-catenin), and vascular endothelial growth factor (VEGF). These interactions activate key pathways, including PI3K/AKT and MAPK/ERK, which collectively inhibit apoptosis, promote cell proliferation, and enhance DNA synthesis, driving hair growth. Arrows indicate signaling flow, with arrow heads indicating stimulation and flat heads indicating inhibition. Red arrows indicate key paths in hair growth.

IGF-1 interacts with a number of other growth factors, as illustrated in Figure 2. While PI3K/Akt is involved in IGF-1 signaling of hair follicle growth via IRS-1, epidermal growth factor (EGF) can activate the same pathway via Src. When combined, these growth factors significantly enhance AKT phosphorylation levels, promoting DNA synthesis and cell proliferation [28]. A transactivation phenomenon also exists wherein IGF-1 activates ERK signaling through EGFR (epidermal growth factor receptor), thus amplifying cell proliferation and apoptosis inhibition signals [29]. EGF activates the RAS/MAPK signaling pathway through EGFR, triggering transition from the telogen phase to the anagen phase and initiating hair follicle proliferation [10,30]. In the HET-1A cell model, a combination of EGF (5 nM) and IGF-1 (10 nM) can increase cell proliferation by 14.6-fold, as compared to 8.2 and 10.4-fold increases for individual growth factor treatments, respectively [31]. This enhanced effect indicated a synergistic interaction between the two growth factors, likely due to phase-specific effects on the cell cycle [31]. IGF-1 is considered a “progression factor,” supporting cell cycle progression, while EGF acts as a “competence factor,” stimulating cell proliferation [30,31]. qRT-PCR analysis shows that their combined action significantly upregulates the expression of hair follicle development-related genes such as LEF1, WNT2, and CCND1 [30]. In human hair follicle culture, the combination also significantly increases Ki67 expression (a proliferation marker) and enhances MAPK/ERK and PI3K/Akt pathway activity [10,30].

IGF-1 also promotes VEGF expression by activating multiple signaling pathways, such as PI3K/Akt and MAPK/ERK, thereby stimulating angiogenesis essential for follicular growth [32]. Together, IGF-1 and VEGF stimulate endothelial cell proliferation, migration, and vascular formation, as observed in human dental pulp stem cells and dermal follicular models [33,34,35]. VEGF-induced angiogenesis promotes anagen phase vascular development and enlarges follicle size, while its expression wanes in catagen and telogen phases [28,36]. Co-administration of IGF-1 and VEGF enhances angiogenesis around hair follicles and reduces hair loss through synergistic effects [37,38,39,40]. Collagen hydrolysates and traditional herbal compounds, such as millet and wheat extracts (MWC), can increase IGF-1 and VEGF mRNA levels [34,38]. Both IGF-1 and VEGF are involved in the Wnt signaling pathway, which plays a pivotal role in hair growth [40,41,42]. Wnt/β-catenin promotes IGF-1 transcription by binding TCF/LEF sites in the IGF-1 promoter [43]. In turn, IGF-1 can enhance Wnt signaling by stabilizing β-catenin and upregulating CCND1, forming a positive feedback loop [44]. β-catenin binding to TCF/LEF also activates the VEGF promoter to promote angiogenesis [44]. IGF-1 stabilizes hypoxia-inducible factor (HIF-1α), a key VEGF regulator [10]. In AGA, the clinical importance of the IGF-1/Wnt/VEGF axis is evident in the lower expression of both IGF-1 and VEGF in dermal papilla cells compared to non-alopecic individuals [10].

Additionally, IGF-1 and TGF-β1 exhibit complex interactions in regulating hair follicle growth. TGF-β1 is a known inhibitor of hair growth and regulates cell differentiation and apoptosis in hair cycling [3]. Exogenous IGF-1 can downregulate TGF-β1 expression in hair follicles in a dose-dependent manner, overriding its suppressive effects and supporting anagen phase maintenance [3].

Keratinocyte growth factor (KGF) activates the MAPK pathway via FGFR2b to stimulate epithelial proliferation. KGF is highly expressed in ORS cells and supports hair follicle regeneration and repair [11]. When combined with IGF-1, it enhances PI3K/Akt and MAPK/ERK pathway activities, promoting hair follicle matrix–cell proliferation and delaying catagen phase onset [37,45,46,47]. Both are primarily synthesized by dermal papilla cells (DPC), which are critical regulators of the hair cycle [45,46,47,48]. IGF-1 and KGF have distinct roles, which may suggest synergistic effects during cell proliferation, as IGF-1 supports hair growth by regulating cell proliferation and migration [46,49], while KGF promotes the proliferation and differentiation of hair follicle epithelial cells [37,45]. As specialized cells located at the base of the hair follicle, DPCs are crucial to the hair growth cycle [50]. Another role of KGF is the determination of hair morphology, as KGF knockout mice display coarse, greasy, and disordered hair that is not corrected by other growth factors [51]. KGF further supports keratinocyte proliferation in both hair follicles and sebaceous glands, and corrects keratinization defects in the hair follicles of nude mice and promotes hair growth [52]. Natural products such as *Koelreuteria paniculata* (varnish tree) and rice bran extracts can modulate IGF-1 and KGF expression by upregulating IGF-1 and KGF mRNA levels in DPCs [46,53]. Similarly to VEGF, KFG expression is abundant in the early anagen phase but decreases over time and is absent in the catagen and telogen phases [51]. KGF expression is androgen-sensitive, and its absence leads to more pronounced hair phenotypes in male mice, suggesting hormonal modulation of its action [51].

IGF-1 also upregulates platelet-derived growth factor-A (PDGF-A) and PDGF-B to promote hair follicle growth and angiogenesis [11]. PDGF binds to its receptor PDGFR and activates RAS/MAPK and PI3K/Akt signaling pathways, promoting cell migration, proliferation, and angiogenesis [54]. When combined with IGF-1, PDGF synergistically enhances Ki67 expression and hair follicle cell proliferation in vitro and in vivo [10,54]. In C57BL/6 mouse models, localized co-injections of IGF-1 and PDGF significantly increased the follicular density in the dorsal skin and promoted earlier entry to the anagen phase [3]. In AGA, the expression levels of IGF-1 and platelet-derived growth factor (PDGF) in dermal papilla cells (DPCs) are significantly reduced compared to those in individuals without alopecia, implicating their deficiency in the pathogenesis of the disease [23,54]. In addition, the bald scalp of AGA patients shows a marked elevation in prostaglandin D2 (PGD2) expression, which has been shown to inhibit hair growth in isolated human hair follicles, with similar inhibitory effects observed upon topical application in mice [55]. Importantly, elevated PGD2 levels may further downregulate IGF-1 expression [56], suggesting that PGD2-mediated suppression of IGF-1 may represent a key mechanism through which AGA negatively regulates hair regeneration.

Other factors also modulate IGF-1 expression. Dihydrotestosterone (DHT) suppresses IGF-1 and impairs follicular proliferation, contributing to androgen-driven hair loss [22]. Conversely, caffeine can stimulate IGF-1 expression, promoting hair growth [57]. Another interesting observation is that exposure to 1763 MHz radiofrequency radiation at 10 W/kg increased IGF-1 mRNA in cultured dermal papilla cells, suggesting potential for non-pharmacological modulation of follicular signaling [12].

## 8. In Vivo and In Vitro IGF-1 Hair Growth Studies

A growing body of preclinical evidence demonstrated the significance of IGF-1 in promoting hair growth and maintaining follicular health, as summarized in Table 1. In multiple C57BL/6 mouse model studies, intradermally injected IGF-1 at different concentrations into dorsal skin significantly increased hair follicle numbers and prolonged anagen phase duration by stimulating matrix–cell proliferation and inhibition of pro-catagenic TGF-β1 [3,11]. Additionally, IGF-1 upregulates the anti-apoptotic protein Bcl-2 while suppressing the pro-apoptotic protein Bax, effectively reducing cell apoptosis and sustaining hair regeneration [11]. This suggests that IGF-1 could be a promising therapeutic target for hair loss treatment. Genetic models further confirm the central role of IGF-1 signaling. In K15-Igf1r conditional knockout mice, loss of IGF-1R in follicular stem cells resulted in a decreased number of dermal papilla cells, abnormal follicular morphology, and reduced expression of BMP-4, a key regulator of folliculogenesis [58]. Interestingly, these mice experienced early entry into the anagen phase but showed delayed transition to catagen, thereby indicating the importance of IGF-1R in coordinating the timing of hair cycle [58]. Conversely, in IGF-1 overexpression mouse models, hair follicle development was accelerated, the anagen phase was prolonged, and hair density was increased [59]. These effects were accompanied by enhanced expression of angiogenic mediators such as VEGF and PDGF-A/B, which improved the follicular microenvironment and sustained hair regeneration [11]. In one study, mice topically treated with 3% IGF-1 gel experienced faster hair growth and thicker hair diameter compared to both the 1% IGF-1 gel and the control groups [60]. In rabbit models, Angora rabbits treated with co-administration of 10 ng/mL IGF-1 and 20 ng/mL EGF significantly enhanced hair follicle cell proliferation and accelerated hair regeneration [30]. In summary, animal model studies showed the complementary roles in follicular stimulation via the anti-apoptotic action of IGF-1 and the cell proliferation mechanism of EGF.

In human hair follicle organ culture models, IGF-1 significantly increased the linear elongation rate of hair follicles at 0.10 mm/day compared to 0.08 mm/day in untreated controls [11]. This effect was attributed to prolonged anagen phase and delayed catagen phase entry by upregulating the anti-apoptotic protein Bcl-2 while inhibiting the pro-apoptotic protein Bax [3,11]. IGF-1 exhibits a well-defined dose-dependent effect on human scalp hair follicles in vitro. Philpott et al. reported that isolated follicles cultured with IGF-1 displayed progressive elongation across concentrations ranging from 0.01 to 100 ng/mL, with maximal stimulation observed at approximately 10 ng/mL and a plateau at higher concentrations [62]. Dysregulation of IGF-1 signaling is implicated in AGA, where microRNA miR-221 overexpression suppresses IGF-1 expression and inactivates the MAPK and PI3K/Akt signaling pathways, leading to hair follicle degeneration [23]. Therapeutic suppression of miR-221 or topical supplementation with IGF-1 has been shown to restore these pathways’ activity, reinitiate anagen, and promote hair regrowth in both preclinical and clinical studies [23,61]. Topical IGF-1 formulations, such as liposomal gels, have been used to treat AGA, resulting in significant improvements in hair density and thickness [61]. The essential role of IGF-1 in follicular development and homeostasis is illustrated in individuals with Laron syndrome (characterized by congenital IGF-1 deficiency), who exhibit sparse and structurally abnormal hair [61]. Similarly, patients with pituitary gland removal or those unresponsive to minoxidil or finasteride may benefit from IGF-1-based therapies as a supplementary or alternative approach [61]. Several studies indicate that endogenous IGF-1 levels can be modulated by lifestyle and nutritional factors. Resistance training, higher dietary protein, particularly from milk and dairy, and certain nutraceuticals such as capsaicin combined with isoflavones have all been reported to increase circulating or dermal IGF-1 [63,64,65].

## 9. Safety and Side Effects

IGF-1 is a potent mitogenic factor that primarily promotes cell proliferation and inhibits apoptosis through the PI3K/Akt and MAPK/ERK signaling pathways. Since the 1990s, IGF-1 has also been administered for short- and long-term treatment of growth hormone insensitivity, during adverse effects including hypoglycemia, lymphoid hyperplasia, benign intracranial hypertension, and coarsening of facial features, were reported; however, these reactions were generally transient [66]. Of note, prolonged IGF-1 stimulation has been associated with cellular senescence through functional interactions with thioredoxin-interacting protein (TXNIP) [67], highlighting the need for further studies to clarify the systemic safety profile of long-term treatment. In addition, during systemic IGF-1 therapy, four cases of benign tumors and one case of malignant tumor have been reported as treatment-emergent adverse events (TEAEs); however, the available data remain insufficient to calculate the relative risk [68].

In contrast, substantial evidence supports the favorable safety of topical IGF-1 ap-plications. In animal models, topical administration of 1–3% liposomal IGF-1 gel did not elevate circulating IGF-1 levels nor induce hepatotoxicity or myelotoxicity, while significantly increasing hair density and shaft diameter, suggesting an absence of systemic toxicity with short-term use [60]. Clinically, in patients with sudden sensorineural hearing loss, local delivery of IGF-1 via gelatin hydrogel not only demonstrated therapeutic efficacy but also showed no serious adverse events during the 16–24 week observation period [69,70]. Mild to moderate events such as tinnitus, dizziness, and otitis media were self-limiting, and no persistent tympanic membrane perforation was observed. Importantly, five-year follow-up data from prior clinical trials further confirmed the absence of tumorigenesis or other late-onset adverse outcomes, providing strong evidence for the long-term safety of topical IGF-1 [70].

Taken together, although IGF-1 has theoretical tumorigenic potential due to its proliferative properties, current animal and clinical evidence consistently indicate that topical application of IGF-1 is well tolerated in both the short- and long-term, without detectable carcinogenic risk. Therefore, IGF-1 can be considered a clinically safe therapeutic option when used locally.

## 10. Future Research Directions

While topical IGF-1 formulations have been used to promote hair growth, their effectiveness has been hampered by the short half-life and low skin permeability. To address these challenges, various methods have been explored to enhance IGF-1 stability and delivery. One such approach involves the creation of a fusion protein by fusing IGF-1 with natural proteins that have longer half-lives, such as Fc proteins, human serum albumin (HSA), or transferrin [71]. These modifications have been shown to extend the in vivo half-life of IGF-1, potentially increasing its efficacy and duration while reducing dosing frequency [71]. Liposome gel delivery systems have been used to demonstrate improved penetration in animal models, resulting in thicker and faster hair growth in hamsters [60]. An emerging field is the use of plant-derived exosomes, such as those derived from ginger, garlic, and *Centella asiatica*, to further optimize IGF-1 delivery by promoting transdermal transport and enhancing uptake by follicular keratinocytes to promote hair growth [72,73]. The biocompatible and naturally sourced exosomes can improve bioavailability and enable targeted delivery to hair follicular cells.

The critical roles of microRNAs (miRNAs) in regulating IGF-1 have been previously noted, and future research could further elucidate the interactions between specific miRNAs and IGF-1 to develop novel therapeutic strategies for hair regeneration. For instance, miR-221 has been shown to suppress IGF-1 gene expression, thereby inhibiting hair growth by impairing dermal papilla cell function. In contrast, miR-218-5p promotes the transition of hair follicles from the telogen (resting) phase to the anagen (growth) phase, enhancing their regenerative potential. Inhibitors (anti-miR/LNA/sponge) can be used to suppress the overexpression of miRNA-221, or mimics (mimic/agomir/exosomes) can be applied to replenish deficient miRNA-218-5p, thereby modulating their regulatory effects on target genes. A deeper understanding of the regulatory networks and crosstalk between these miRNAs and IGF-1 could offer significant insights and advances in the development of effective treatments for hair loss. [23,74]. Moreover, combinatorial strategies involving IGF-1 and other growth factors (e.g., VEGF, FGF-7, PDGF) also warrant further exploration. Preliminary studies suggest synergistic effects on follicular proliferation, angiogenesis, and anagen extension, indicating that multi-factor therapies could be preferable over monotherapies. Understanding the crosstalk between IGF-1 and these signaling networks will be essential to designing personalized, mechanism-based treatments for various forms of hair loss.

## 11. Conclusions

IGF-1 is critical in promoting hair growth with roles in stimulating follicular cell proliferation, extending the anagen phase, and preserving follicle integrity through anti-apoptotic mechanisms [3,10,11,23]. Its clinical relevance in androgenetic alopecia and alopecia areata has been demonstrated by topical or targeted delivery of IGF-1 to increase hair density and improve follicle structure [23,61].

IGF-1 exhibits synergistic potential when combined with other growth factors such as VEGF, FGF-7, and PDGF, activating complementary pathways to enhance follicular resilience and regeneration that may serve as a basis for next-generation combination therapy tailored to individual patient needs [75]. Such personalized treatment plans may also integrate diagnostic approaches, including miRNA profiling and customized delivery systems, solidifying the commercial value and market potential of IGF-1 as a basis for innovative hair restoration solutions [61,76]. IGF-1 is a promising industry benchmark for optimizing hair regeneration outcomes as future research progresses in refining molecular mechanisms, optimizing therapeutic combinations, and developing innovative delivery systems.

## Figures and Tables

**Table 1:** Research on IGF-1 and Its Effects on Hair Growth in in vivo Model.

Model	Function	Reference
C57BL/6 mice	Local injection of IGF-1 increased hair follicle number and prolonged the growing phase during the transition from anagen to telogen.	[3]
K15-Igf1r(KO) mice	K15-Igf1r(KO) HFs entered anagen phase earlier than controls and showed a delay in the anagen/catagen switch. The expression of Bmp-4 mRNA was inhibited in HFs from K15-Igf1r(KO).	[58]
Adult HK1.IGF-1 mice	HK1.IGF-1 transgenic mice developed papillomas faster and in markedly greater numbers compared to non-transgenic littermates.	[59]
Hamsters	Efficacy was determined by dermatoscopy analysis of hair density and microscopy analysis of hair diameter, with hair found to be thicker and with more rapid growth in the 3% group than in either the 1% group or the control group.	[60]
Angora Rabbit	The IGF-1 and EGF combination promoted the transition of the hair cycle from telogen to anagen and stimulated the growth of hair shafts. This IGF-1 and EGF combination maintained the structure of the HF and enhanced the cell proliferation of outer root sheaths and the dermal papilla within rabbit skin.	[30]
Human	miR-221 inhibited hair growth and the proliferation of dermal papilla (DPCs) and sheath cells (DSCs) in androgenetic alopecia (AGA) patients, correlating positively with AR expression and negatively with IGF-1 expression.	[23]
Human	Dermal papilla cells from balding scalp follicles secrete significantly less IGF-1 compared to those from non-balding scalp follicles.	[61]

## Abbreviations

The following abbreviations are used in this manuscript:

AGA	Androgenetic alopecia
EGF	Epidermal Growth Factor
EGFR	EGF Receptor
IGF-1	Insulin-like Growth Factor 1
IGFBPs	IGF-Binding Proteins
IGF-1R	IGF-1 Receptor
IRS1/IRS2	Insulin Receptor Substrates 1/2
KGF	Keratinocyte Growth Factor
PDGF	Platelet-Derived Growth Factor
PDGFR	PDGF Receptor
PIP2	Phosphatidylinositol 4,5-Bisphosphate
PIP3	Phosphatidylinositol 3,4,5-Trisphosphate
TGF-β1	Transforming Growth Factor-β1
VEGF	Vascular Endothelial Growth Factor
